# Alkalis in Rheumatism

**Published:** 1855-03

**Authors:** George Budd

**Affiliations:** Physician to King’s College Hospital


### THE USE OF ALKALIS IN ACUTE RHEUMATISM.

BY GEORGE BUDD, M. D., F. R. S.,

Physician to King's College Hospital.

One of the last lectures of Dr. Budd, on Acute Rheumatic Fever, seems to us of unusual interest, the treatment of this disease, as we have observed it at King's College Hospital, being so satisfactory and novel. Dr. Budd, after going into a description of the ordinary phenomena of rheumatic fever and rheumatism, dwelt on one of the more formidable and common results of rheumatic fever, namely, diseased heart.

"The frequent occurrence of this complication of rheumatic fever was now so well known, indeed, as to require only to be stated. He thought, in one half of the cases—nay, more—three-fourths of the cases of rheumatic fever coming into King's College Hospital, they found severe disease of the heart. It may occur," said Dr. Budd, "at the onset, or still later in the disease; but as a general rule, it will be found to run parallel, so to speak, with the fever and constitutional derangement. We find the deposit from the blood or inflammatory result under two chief forms: first, and most formidable, deposits of lymph—beads of lymph, so to speak, on the edges of the valves of the heart, of the left side particularly, impeding more or less its normal functions. These effusions, or beads of lymph, are the result of a peculiar increase in the fibrinous portions of the blood. These deposits give us the stethoscopic signs of diseased valves. We have next, effusion into the sac of the pericardium, with so-called pericarditis: and very often also inflammation of the contiguous pleura. Now I think, if you will watch the cases in the wards," continued the lecturer, "these of Dr. Todd and mine, that you will find at least three or four cases of rheumatic fever, with diseased valves and bellows-murmur; to one case of deposit on the exterior of the heart with friction-sounds. This is a very useful and practical point to keep in mind. Any disease of such delicate parts as the valves must be more serious, as leading to permanent organic disease, and cannot too soon engross our attention.

"Now, as to rheumatic fever, what do we generally find? Very frequently you will not discover all that is in books; but pain and palpitation, if enquired after, are generally found; pain of an obscure, dull character, over the region of the heart; there is also that remarkable hurry of breathing, which betokens fever; in place of the respirations numbering as they should number, about 18 to 20, they are higher,—30, or even double the normal amount —when the valves take on the disease. With this rapidity of breathing we have what is called a single bellows-sound with the first sound of the heart; in other words, a systolic *bruit*, very well marked. This sound, in contradistinction to the friction or rubbing-sound is best heard at the apex of the heart, the rubbing-sound ending, and very possibly will be heard only for two or three days, and then ceases when adhesion takes place, the valvular *bruit* still audible at the apex. The point I would next wish to draw your attention to, is the great tendency to relapse observed in rheumatic fever. The patient, the chances are very many, will tell you he has had rheumatism before—aye! may be two or three, or even four times. Rheumatic fever, it must be confessed, is a very obscure disease; it is more common in London than in the eastern parts of England. It is evidently modified by climate; it is more common amongst men than women, it seldom occurs beyond the age of 30, the chief tendency to the disease existing between the age of 15 and 30. Some persons of a thin, ligamentous development of body are peculiarly susceptible of it, and the most frequent cause seems to be damp combined with cold.

"We now come to the essential question of *Treatment*. This at King's College Hospital, after treatment of various kinds, I find to be best under the form of large doses of alkalis. I usually prescribe the bicarbonate of potash (gr. xii ad gr. xv,) with the nitrate of potash (gr. v,) every four hours; or if we put it in technical language, it will be .

| | | |
|---|---|---|
| ℞ | Potassæ bicarb. | one drachm, |
| | Potassæ nit. | " scruple. |

Tere simul bene et in part. iv divide; detur i hora quarta quaque. I know no plan of treating acute rheumatism at all equal to this; it suggested itself to us from the large amount of acid in the system. I think it as successful, if not more so, than any other plan tried in the University College Hospital. It is quite as remarkable how the symptoms yield according as the urine becomes alkaline. You will find, where many joints are affected, that the urine is extremely acid. You will do well to keep the bowels also well opened, as mal-assimilation assists the rheumatic diathesis; colocynth extract, and a little blue-pill, or a saline cathartic mixture, according to circumstances, should be prescribed. Another medicine of great value in rheumatic fever, and one which you cannot do without, is opium; you will find your patient with rheumatic fever gets worn out if you do not give him a moderate draught containing morphia or opium at night. You must take care and economise your patient's strength; take care he is not worn out, for in all such patients rheumatic fever is much more difficult to cure. I find it necessary to keep up strength very often, and then we order a mixture of decoction of cinchona and the alkaline carbonates as before,—a mixture perhaps not very chemical, but still very useful: indeed, most eminently so, in restoring the "tone" of the system as the appetite and strength.

"There is another subject now on which I wish to speak—namely, *local* treatment; this is a point, perhaps not sufficiently attended to: it has been found that the joints of the body most exposed, such as the wrist, ankle, knee, &c., are more liable to rheumatic inflammation than the shoulder or hip well covered with muscles. Accordingly, it is found useful to take the hint, and in this hospital we envelope the limb in oil silk, and cotton wool; we also find a warm alkaline fomentation—half an ounce of carbonate of potash to a pint of water gives very considerable relief. There is another application I have great faith in—a small blister, not placed *on* the joint, but *above* it, between the joint and the heart; it seems to act by drawing off the inflammation from the joint to the parts above, so as not to be aggravated by motion—mind the blister is *above* the joint, not at all over the joint. A blister may be said to be lowering, but rheumatic inflammation is much more lowering; finally, if there should remain chronic thickening of joints, I order the iodine paint. I will now read for you two or three cases out of the hospital book, just gone out cured, illustrative of what I say, and then speak of diagnosis. And, first, from gout—in gout there is more effusion—the skin also is more darkly red, almost mahogany-color. Gout proverbially attacks a different class of persons, chiefly above thirty years of age, the *bon-vivant.* We have two cases, however, of gout now in hospital, but one is a man who has had delirium tremens over and over again. There is another disease, viz., gonorrhœal rheumatism; here one or more joints are affected, but you will find less fever, pulse not so high; you will find it also a most protracted and troublesome disease; it may last for three or four months. Again, you must not mistake syphilitic periostitis; the pains here are not in the joints but in the shafts of the more exposed bones, with nodes and other chronic secondary symptoms. Iodide of potassium is the chief remedy. I know no remedy, however, for gonorrhœal rheumatism,—perhaps a blister above the joint is best with extensive discharge, cut off the scarf skin of your blister, and dress the latter with green ointment; in ordinary rheumatism, however, I should not, nor do I ever, remove the scarf skin, but dress the blisters—two, or three, or four perhaps with simple cerate. I mention these few points, as really a very great deal depends on them.—*Medical Circular.*

